# Triage May Improve Selection to Colonoscopy and Reduce the Number of Unnecessary Colonoscopies

**DOI:** 10.3390/cancers12092610

**Published:** 2020-09-12

**Authors:** Mathias M. Petersen, Linnea Ferm, Jakob Kleif, Thomas B. Piper, Eva Rømer, Ib J. Christensen, Hans J. Nielsen

**Affiliations:** 1 Department of Surgical Gastroenterology, Hvidovre Hospital, 2650 Hvidovre, Denmark; mathias.mertz.petersen@regionh.dk (M.M.P.); linnea.ferm@regionh.dk (L.F.); jakob.kleif@regionh.dk (J.K.); thomas.baastrup.piper@regionh.dk (T.B.P.); Eva.Christine.Aastrup.Roemer@regionh.dk (E.R.); ib.jarle.christensen@regionh.dk (I.J.C.); 2 Institute of Clinical Medicine, University of Copenhagen, 2100 Copenhagen, Denmark

**Keywords:** early detection, screening, triage, biomarkers, feces testing, colonoscopy, colorectal cancer

## Abstract

**Simple Summary:**

Presently, constraints on colonoscopy capacity appear to be associated with inclusion of screening by direct colonoscopy or follow-up colonoscopy subsequent to a positive result of a feces-based screening concept. It is well known, however, that only a minority of the subjects with a positive feces test are diagnosed with bowel neoplasia at the subsequent follow-up colonoscopy. Therefore, a proposed Triage test concept, which includes (1) age of the subject; (2) concentration of occult blood in a feces test; (3) combinations of blood-based, cancer-associated biomarkers, may improve selection to follow-up colonoscopy in screening for bowel cancer. Thereby, the number of unnecessary colonoscopies may be reduced significantly, which may improve the national healthcare budgets, and indeed spare many subjects for the colonic examination, which is not free from adverse effects. Current research may identify and validate the optimal Triage screening concept.

**Abstract:**

Implementation of population screening for colorectal cancer by direct colonoscopy or follow-up colonoscopy after a positive fecal blood test has challenged the overall capacity of bowel examinations. Certain countries are facing serious colonoscopy capacity constraints, which have led to waiting lists and long time latency of follow-up examinations. Various options for improvement are considered, including increased cut-off values of the fecal blood tests. Results from major clinical studies of blood-based, cancer-associated biomarkers have, however, led to focus on a Triage concept for improved selection to colonoscopy. The Triage test may include subject age, concentration of hemoglobin in a feces test and a combination of certain blood-based cancer-associated biomarkers. Recent results have indicated that Triage may reduce the requirements for colonoscopy by around 30%. Such results may be advantageous for the capacity, the healthcare budgets and in particular, the subjects, who do not need an unnecessary, unpleasant and risk-associated bowel examination.

The aims of population-based screening for colorectal cancer (CRC) include improvement of cancer-specific survival [1] and reduction of the incidence of the disease [2], which ultimately may reduce the prevalence. Globally, a variety of screening concepts are either used or are under evaluation and subsequent validation [3]. The accepted current concepts include direct colonoscopy [3] and screening using fecal immunochemical tests (FIT) for occult hemoglobin in feces [3,4]; a positive FIT result leads to recommendation of subsequent follow-up colonoscopy [4,5]. The sensitivity for detecting CRC lesions by direct colonoscopy appears to be 95%, and FIT has a sensitivity around 76% [6]. While CRC detection by direct colonoscopy is largely independent of the stage and location, detection by FIT screening is highly T-stage- and location-dependent [6,7,8]. Specifically, the sensitivity for detection of T1 lesions is limited in comparison with higher T-stages, and in addition, detection of T1 lesions appears to be highly dependent on location, even with application of various FIT cut-off values [8]. FIT detection of advanced adenomas may be even more dependent on the location, ranging from 0% of caecal lesions, to 26% of ascending colon and right flexure, 14% of transverse colon and left flexure, to 58% of descending colon and 51% of sigmoid lesions, while 36% of rectal lesions appear to be detected [9]. 

The outcome of screening for bowel neoplasia is dependent of the test concept and the test sensitivity, but the major challenge appears; however, to be the limited compliance rates [10,11,12]. In addition to subject compliance the various specific healthcare systems have influence on population screening, ranging from insurance- or self-paid screening in some countries, mostly in the USA, to community-paid screening in other countries, mostly in Europe. Thereby, the compliance rates in colonoscopy screening are income-dependent in the USA, where economy also plays a significant role in FIT screening and may limit the follow-up colonoscopy rate of those with a positive FIT result. Many American citizens, specifically uninsured with a positive FIT result, are far from instantly undergoing the recommended follow-up colonoscopy [13,14], and the lead-time has been shown to be associated with increased risk of CRC and higher stages at final diagnosis [15,16]. Even among subjects with access to screening free of charge, the colonoscopy compliance rates are far from close to 100% [17,18]. In Denmark, where current legislation dictates that the lead-time from a positive FIT test to a subsequent follow-up colonoscopy has to be less than 14 days, the colonoscopy compliance rates are still suboptimal, at around 90% [19]. Such results underline that lack of follow-up colonoscopy is not only based on economy, but certainly, other issues including subject decline, social barriers and comorbidity may play an additional, significant role [12,13]. 

Subsequent considerations include the efficacy of the screening test to detect subjects with CRC in the various populations. While the efficacy of direct colonoscopy screening is close to optimal, the FIT screening is less effective, because of the determinant compliance limitations. In Denmark, FIT screening roughly detects 62% (compliance) × 76% (test sensitivity at cut-off 100 ng/mL, Table 1) = 47% of the subjects, who in the screening-relevant age (50–74 years) may have CRC [20]. Therefore, both sensitivity and compliance of the test are specific areas that need utmost combined attention to improve the overall outcome of colorectal cancer screening of the average risk population. Although limited compliance to feces-based testing programs appears to include feces aversion, personal and social issues, it may also be a matter of serious considerations in the event that the test is positive and thereby leads to recommendation of a possibly unpleasant follow-up colonoscopy that includes specific discomfort during bowel preparation [21]. The latter argument may be supported by the previous extremely limited annual compliance to screening colonoscopy of only 2.6% in Germany, where the regional healthcare authorities paid for the procedure [22]. Future screening concepts may focus on acceptability by the screening-relevant population. One such option may be screening based on various cancer-associated biomarkers in blood samples; emerging results have indicated that blood-based screening may improve the screening test sensitivity [23,24,25,26,27,28], in particular by combining various biomarker entities [29]. Blood-based screening appears to be preferred in comparison with feces-based testing [30,31], although the feces test performance is still the single most important attribute of a screening test [31].

Hitherto, the majority of emerging results on blood-based, cancer-associated biomarkers for early detection of malignant diseases, including CRC, have been based on studies of symptomatic patients at risk of having the disease or on patients with a diagnosis of the disease or even a mix of symptomatic and diagnosed patients [23,24,25,26,29,32]. It has been widely discussed whether such results might be interpreted to be used directly in screening settings. Although recent achievements have shown that results generated on blood samples collected from minor studies of screening for CRC by direct colonoscopy may have a certain value [33,34], there may be significant discrepancies between results generated in blood-samples from symptomatic patients and subjects undergoing colonoscopy screening [35]. Emerging achievements underline that there are major and significant discrepancies between results generated from symptomatic patients and from subjects undergoing current established screening [36,37]. The results from one study highlight that the discrepancy is huge and cannot be neglected, because 2 × > 4000 subjects with clinical results confirmed by colonoscopy were included in the comparison. The discrepancy may be well explained based on the current achievements, which show significantly higher levels of cancer-associated protein biomarkers among symptomatic subjects compared to screened subjects [37]. Although future research on biomarker discovery, which may have focus on symptomatic subjects or even patients with a final malignant diagnosis is still acceptable, subsequent training and validation need to concentrate on sufficiently sized and well-performed screening studies [38].

Focus on FIT screening results and emerging results from blood-based biomarker studies indicate that FIT screening has some location- and T stage-associated limitation in detecting neoplastic lesions [20], while blood-based biomarkers may have location-independent limitations with the T1 lesions of CRC and some adenomas [37]. Future achievements for improving CRC screening may therefore consider combinations of FIT and blood-based biomarkers [39]. Specifically, it may be considered that the combination identifies additional subjects with neoplastic lesions in comparison with the separate FIT or blood-based screening test, respectively. Recently, the combination of feces-based DNA and FIT showed that combinations of FIT with other entities may improve detection sensitivity [40]. Combined attitudes may be the basis for improved selection to colonoscopy and may thereby play a significant role in harmonizing the hugely different cut-off levels and screening age intervals between different national programs. Specifically, The Netherlands, which initiated FIT screening with a cut-off level of 17 µg Hb/g feces, which corresponds to 85 ng Hb/mL buffer, has realized that the colonoscopy demand was too large and therefore had to increase the cut-off level to 235 ng/mL [41,42]. Many European countries, which are performing screening in parts of their country or are still considering initiating universal population screening using the FIT test, have preliminary chosen a high cut-off level to reduce the well-known high requests on colonoscopy (Table 1). 

**Table 1:** Cut-off levels for FIT CRC screening in various European countries.

Country	Cut-off Level	Screening Age Interval [Reference]
Denmark	100 ng/mL	screening age interval: 50–74 years [19]
France (Paris)	150 ng/mL (30 µg Hb/g)	screening age interval: 50–74 years [43]
The Netherlands	235 ng/mL (47 µg Hb/g)	screening age interval: 55–75 years [41]
Sweden (females)	200 ng/mL	screening age interval: 60–69 years [44]
Sweden (males)	400 ng/mL	screening age interval: 60–69 years [44]
Scotland	400 ng/mL (80 µg Hb/g)	screening age interval: 50–74 years [45]
England	600 ng/mL (120 µg Hb/g)	screening age interval: 60–74 years [46]
Wales	750 ng/mL (150 µg Hb/g)	screening age interval: 60–74 years [47]

Despite establishing such high cut-off levels, in some countries the wait-time for colonoscopy still appears to be significant [48]. Recent calculations showed that increase of the cut-off level from 100 ng/mL to 200 ng/mL would reduce the colonoscopy requirements with some 32% [49,50]. Therefore, the high cut-off levels chosen by some countries may alleviate some of the colonoscopy burden, but unfortunately, the costs for increased cut-off levels are that high numbers of significant neoplastic lesions, including CRC, will be missed [8,49,50].

Definitely, every single country that initiates population screening may face significant increased requirements for colonoscopy, not only for follow-up procedures, but certainly also for adenoma control; in some countries, the amount of adenoma control colonoscopies accounts for up to 25% of the total numbers [51]. Ultimately, the numbers of screening associated colonoscopies were expected to reduce the numbers of diagnostic colonoscopies of subjects with symptoms attributable to CRC, but due to changed legislation in some countries, symptomatic subjects are to be offered examination, including colonoscopy, within a few weeks. Therefore, the numbers of diagnostic colonoscopy do not seem to be reduced. It is well-known that far from all colonoscopy procedures are needed; only some 35–40% of the subjects with a positive FIT result have bowel lesions (CRC, high-risk adenoma, or medium-risk adenoma) [52], less than 20% of the subjects undergoing adenoma control colonoscopy have new lesions [51] and only 25–30% of subjects undergoing colonoscopy due to symptoms have neoplastic lesions [23,53]. Combination of these figures show that the amount of unnecessary colonoscopies performed appears to be between 60% and 80%, and the current arguments that increased cut-off levels may restrict the procedure to those with the highest risk of having a significant neoplastic lesion is somehow contradicted by the fact that around 50% of the subjects with 1000 ng/mL (upper detection limit, OC-Sensor) in the FIT test do still not have CRC (Figure 1, solid red line).

Due to present constraints with colonoscopy capacity specifically in relation to FIT-based screening, which has already been implemented or is under consideration for implementation, we need to focus on improving the selection criteria for colonoscopy. One such opportunity is further validation of the Triage test suggested previously [49,50]. That specific test includes (1) the age of the subject; (2) the level of hemoglobin in the FIT test; and (3) combined biomarkers, which may be a mix of proteins and ctDNA methylations, mutations and/or fragmentations (Figure 1) [23,24,25,26,27,28,29]. Particularly, the various possibilities for biomarker combinations require a high level of research attention to develop, test and validate the combinations with the highest and most reproducible impact. This may also include miRNA, nucleosomes, histone modifications, glycoproteins, autoantibodies, etc. [38], but it must be stressed that the combined blood-based tests need to be simple, reliable, cost-effective and suited to be determined using automated analysis platforms to get results at a high quality level within shortly. Preliminary results indicate that the Triage concept may reduce the numbers of unnecessary colonoscopies with some 32% [49,50]. Thereby, a significant proportion of the subjects, who would be offered an unnecessary bowel examination, will be spared the unpleasant and far from effective bowel preparation, which may even hinder adequate intra-luminal examination [54,55]. In addition, it has to be emphasized that colonoscopy is associated with subjects being out of work or daily routines for 1–1½ days [20]; the screening age intervals cover a significant part of subjects in the active work force. Finally, a plethora of recorded, under-recorded and non-recorded adverse events associated with colonoscopy and ranging from post-procedure cognitive impairment through cardiopulmonary incidents and bleeding episodes to perforation, septic complications and ultimately death [56,57,58,59] would be reduced.

It has been argued, however, that the costs of the Triage test may exclude any attempt at further validation and even implementation. Against such arguments stands the costs for unnecessary colonoscopies, which by reduction of > 30% would more than support Triage being an option to improve selection to colonoscopy. Indeed, the pressure on the various healthcare budgets may be improved with subsequent room for considerations of extension of the screening age intervals and even lowering the cut-off levels significantly (Table 1). In addition, improved selection may underline the necessity for every single subject to accept the follow-up colonoscopy procedure in the event of a positive Triage test.

Recently, it was shown that the number of patients with young-onset colorectal cancer appears to increase [60,61,62]. Those uniform, globally-based results underlining an ongoing increase of the incidence led to suggestions of lowering the onset screening age to 45 years of age [63,64,65]. Presently, the American Cancer Society (ACS) has adopted the evidence and recommended screening age to start at the age of 45 years [66]. The United States Preventive Services Task Force (USPSTF) is still recommending screening age to start at 50 years but is presently working on new recommendations to be released shortly [67]. Both ACS and USPSTF also recommend screening of subjects in the age range of 76–85 years; the decision to screen for colorectal cancer in adults of that age should be individual and need to take the subject’s preferences, life expectancy, overall health and prior screening history into account. Subjects in such high age ranges need to have the ability to undergo treatment, including surgery in the event of neoplastic findings at screening.

Taking the well-known constraints of colonoscopy procedures into account it is needed to state that direct colonoscopy screening for entire populations will not be a possibility in most countries; such procedures are reserved for subjects with insurances or those that are paying privately. In Europe we need to consider population screening of entire populations based on the FIT testing concepts; it was recently shown that the cost-utility is better for FIT screening compared with colonoscopy screening [68]. As suggested above, addition of a blood-based, cancer-associated biomarker test to improve selection to colonoscopy may indeed improve the entire selection to colonoscopy, both for screening follow-up, adenoma control and diagnosis of subjects with symptoms. With focus only on follow-up colonoscopies, we need to consider the consequences if screening within Europe has to be recommended for subjects between 45–85 years of age. It may be added to the current considerations that screening is recommended to start at the age of 40 years for subjects, who are first-degree relatives to a patient with CRC or with advanced adenoma in the age < 60 years [69]. In addition, recent research results even recommend screening age to start at 40 years for the average-risk population [70].

In conclusion, recent achievements and current research results underline that the Triage concept may lead to improved selection to colonoscopy. Definitely, an improved selection will be a significant achievement not only for the present colonoscopy constraints with long wait times for the procedure and for the healthcare budgets, but specifically for those subjects, who do not need to undergo an unnecessary and unpleasant bowel preparation and subsequent examination, which is not even free from side effects. Ultimately, such improved selection criteria may also increase the number of those, who will agree to the subsequent follow-up colonoscopy when screened positive. At present, results from two major clinical Triage-based studies with focus on subjects undergoing population screening and subjects offered colonoscopy due to symptoms attributable to colorectal neoplasia, respectively, are awaited with interest.

## Figures and Tables

**Figure 1:**
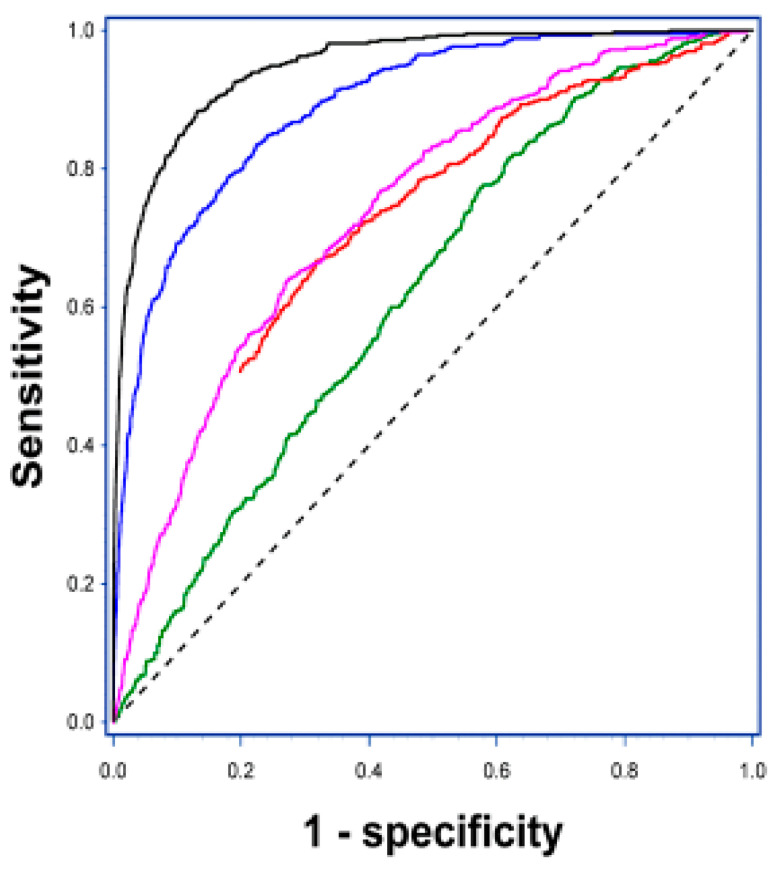
Illustration of the Triage concept, which includes age of the subject, the FIT hemoglobin concentration and various blood-based, cancer-associated biomarkers. ROC curves for: age of the subjects (green), FIT hemoglobin concentration (red), combined age and FIT hemoglobin concentration (magenta), addition of blood-based protein biomarkers (blue) and addition of ctDNA methylations (black). The curves are based on results from a current, major study ([38], age and FIT results) and a simulation model based on independent protein and ctDNA methylation biomarkers. The FIT hemoglobin level is cut at 1000 ng/mL, which is the highest level of detection (OC-Sensor). Extrapolation into the y-axis shows that only 52% of the subjects with 1000 ng/mL have CRC.
